# Integrity of personal radiation protective equipment (PRPE): a 4-year longitudinal follow-up study

**DOI:** 10.1186/s13244-022-01323-3

**Published:** 2022-12-06

**Authors:** Pieter-Jan Kellens, An De Hauwere, Tim Gossye, Sven Peire, Ingrid Tournicourt, Luc Strubbe, Jan De Pooter, Klaus Bacher

**Affiliations:** 1 Medical Physics, Ghent University, Proeftuinstraat 86, 9000 Ghent, Belgium; 2 AZ Sint-Jan Brugge - Oostende AV, Brugge, Belgium; 3 Heart Centre, University Hospital Ghent, Ghent, Belgium

**Keywords:** Radioprotection, Quality assurance, PRPE, Integrity defects

## Abstract

**Background:**

Personal radiation protective equipment (PRPE) such as lead aprons minimises radiation exposure of operators using X-ray systems. However, PRPE might be prone to cracks in the attenuating layer resulting in inadequate radiation protection. This study aims to investigate the prevalence, qualification and quantification of PRPE integrity during a longitudinal follow-up study.

**Methods:**

All PRPE of a large, general hospital was evaluated yearly in the period 2018–2021. The equipment was inspected on a tele-operated X-ray table, and tears were qualified and quantified using an X-ray opaque ruler. Rejection criteria of Lambert & McKeon, with an extra rejection criterion of 15 mm^2^ for individual tears, were applied to accept or reject further use of the PRPE.

**Results:**

Over the 4-year follow-up period, a total of 1011 pieces of PRPE were evaluated. In total, 47.3% of the PRPE showed tears of which 31% exceeded the mentioned rejection criteria. Remarkably, of the 287 newly registered pieces of PRPE, 6.0% showed tears in the first year of use of which 88.2% needed to be rejected. Also, 48% of the repaired PRPE was rejected again in the consecutive year.

**Conclusions:**

PRPE is prone to cracks. Up to 50% of PRPE showed tears and cracks resulting in 31% rejections. Newly purchased PRPE is not guaranteed to remain free of cracks and tears in the first year of use. Repair does not guarantee a long-term solution for prolonging the lifespan. Regular X-ray-based integrity analysis of PRPE is needed to ensure adequate radioprotection for operators using X-ray systems.

## Key points

1. PRPE are susceptible to hidden integrity defects.
2. Regular quality control is needed to ensure adequate radiation protection.
3. New and repaired pieces are not guaranteed to remain free of tears.

## Background

Fluoroscopically guided procedures expose medical staff members to ionising radiation and could impose staff to increased health issues [1–3]. Besides essential radiation safety measures such as maximising distance and minimising exposure time, personal radiation protective equipment (PRPE) further reduces exposure to ionizing radiation [3–5].

PRPE consists of lead aprons, vests, skirts and thyroid shields which initially contained lead powder-loaded sheets or lead-oxide on vinyl [6–8]. However, due to the weight and environmental issues, there was a shift towards lightweight, non-lead or lead-composite gear [8–12]. Besides millimetre lead equivalence, a reference unit for the degree of radiation protection corresponding to a thickness pure lead, the protective features depend on the condition of the attenuating layer. PRPE could be sensitive to wear and tear resulting in cracks and tears. Unfortunately, analysis of PRPE integrity remains very scarce. Bawazeer et al. describe a large sample size of annual inspection data of PRPE, though based only on compiled inspection data of one year hence only revealing snapshot information [13]. Longitudinal research on a large sample size has been performed by Oppliger-Schäfer et al. at the University Hospital of Basel, yet remains rather limited in tear analysis [14].

To the best of our knowledge, our study is the first to analyse PRPE integrity in clinically used PRPE in a longitudinal follow-up of cracks in the attenuating layer.

## Methods

### Aim

This study aims to investigate the prevalence, qualification and quantification of PRPE integrity during a longitudinal follow-up study.

### Study design

All PRPE of a general hospital were assessed yearly during longitudinal follow-up study from 2018 to 2021. The following departments participated: pain clinic (PC), cardiology, surgery theatre (ST), medical imaging (MI) and gastroenterology, oral and maxillofacial surgery and urology (G/MKA/URO). Every piece was labelled with a unique identification number (TN) to allow for longitudinal follow-up. For all PRPE of the participating departments, size, type, brand, manufacturing date and lead equivalency were registered. If the piece of PRPE had an individual name tag, this was recorded as well. The five most frequently used brands were Brand A (Infinity), Brand B (Tema), Brand C (MDT), Brand D (Scanflex) and Brand E (Protec X). Brands A and B allowed the repair of tears and cracks except for thyroid shields. Brands C, D and E did not allow repair. A PRPE integrity analysis was performed by medical physics experts (authors P.K., A.D.H., T.G.) on behalf of the health and safety department of the hospital.

### PRPE integrity analysis

#### Visual inspection

PRPE first underwent a visual inspection. This included examination of the closure of the buckles, hook and loop fasteners and magnets as well as inspection of the outer fabric. Any damage to the exterior surface was reported in the logbook for each piece of PRPE.

#### X-ray-based integrity analysis

An X-ray-based integrity analysis was used to assess the integrity of PRPE. The equipment was scanned fluoroscopically with a Siemens Luminos dRF (Siemens Healthineers) according to a standardised protocol. This protocol consisted of an abdomen anterior–posterior protocol with automated brightness control. A field size of 42 cm × 42 cm and a source-to-image distance (SID) of 115 cm were used. Defects, visible as white surfaces in fluoroscopy mode, were recorded and quantified using an X-ray opaque grid with an interspace of 1 cm. The size of the defects was estimated based on this grid. In situations where defects were suspected, a tennis ball was laid underneath to quantify more easily (Fig. 2).

#### Rejection criteria

The applied centre’s rejection criteria were based on the criteria of Lambert and McKeon [15]. Pieces with a total defect area larger than 670 mm^2^ were rejected. In addition, an institutional rule applied that a single tear of more than 15 mm^2^ would also result in rejection. In exception, thyroid shields were rejected when the total defect area exceeded 11 mm^2^ or a single tear exceeded 5 mm^2^.

#### Statistical analysis

Data and statistical analyses were performed in Rstudio (Rstudio, Boston), using a dedicated data science package tidyverse in R (team Rc, Austria) [16]. Summary statistics for continuous variables are given as median ± interquartile range (IQR) or mean ± standard deviation (SD). Percentages are shown with their absolute values in parentheses. Logistic regression was used to inspect the association between outcome variable ‘test result’, and predictor variables ‘PRPE brand’, ‘test year’ and ‘department’. A comparison of multiple groups was visualised using sina plots. Compared to boxplots, a sina plot has the benefit that the entire distribution is shown as a kernel density plot besides the quantile descriptive statistics. This symmetric plot has wider sections that correspond to a higher probability and vice versa. All data points were (log_e_(mm^2^) + 1)—transformed to enhance visualisation because of the highly right-skewed character of the distribution. The + 1 inside the logarithm was an arbitrarily chosen constant in order to include pieces with a tear area of 0 mm^2^. A comparison of multiple continuous groups was performed with two-sided, nonparametric Kruskal–Wallis test. Dunn’s post hoc test was used to control the family-wise error rate (FWER) at a 5% significance level. The location-shift model assumption was fulfilled allowing us to express the alternative hypothesis in terms of means.

## Results

### Overview of PRPE

Overall, 1011 unique pieces of PRPE (individual TN) were assessed. Every piece was assessed once a year resulting in a total of 2588 quality checks. More specifically, 856 thyroid shields, 695 vests, 649 skirts and 388 aprons were inspected. A total of 613, 583, 659 and 733 controls were evaluated in 2018, 2019, 2020 and 2021, respectively. All pieces were 0.25 mm lead equivalent with a wrap-around fit to provide 0.5 mm lead equivalent protection at the front side. Exceptions were thyroid shields and older pieces consisting only of front protection. Both were 0.5 mm lead equivalent.

The number of unique pieces of PRPE evaluated four times was 30.4% (307), while 22.6% (228) was evaluated three times, 19.8% (200) was evaluated two times, and 27.3% (276) was evaluated only once. The number of pieces of PRPE with a unique name tag was 14.9% (151). Visual inspection revealed that 15% (387) of the controls had visual defects. These included detaching hook and loop fasteners, broken buckles, exfoliation and glueing of the outer fabric and torn seams revealing the attenuating layer.

### Overall acceptance and rejection rates of PRPE

During the study period, 55% (1422/2588) of the controls revealed tears of which 34% (483/1422) exceeded the rejection criteria. The annual rejection rates were, respectively, from year 1 to year 4, 17.6% (108/613), 17.3% (101/583), 22.3% (147/659) and 17.3% (127/733). This led to an average rejection rate of 18.6% (483/2588). In terms of unique PRPE (individual TN), 47.3% (478/1011) out of 1011 individual TN’s showed a tear at least once of which 31% (148/478) exceeded the rejection criteria. The median tear areas were for, respectively, thyroid shields, skirts, aprons and vests, 14 ± 34 mm^2^, 40 ± 117 mm^2^, 23 ± 64 mm^2^ and 30 ± 69mm^2^.

### Incidence of cracks in PRPE in the first year of use

During our study, 287 new pieces of PRPE from different manufacturers were registered. In the following quality check, 6.0% (17) of those new pieces showed tears. Of those 6.0%, 88.2% (15) exceeded the rejection criteria. The median tear area was 70 ± 60 mm^2^.

### Comparison of repairable and unrepairable pieces

Table 1 displays yearly rejection rates of the five most frequently used brands and rejection rates combined for the other brands. PRPE brand and test result were found to be associated (*p* value < 0.001). The repairable brands (A and B) were significantly higher in rejection rate compared to the other frequent brands (*p* value < 0.001). Predictor variable ‘year’ was found to be independent of outcome variable ‘test result’ (*p* value > 0.05).

**Table 1:** Yearly total rejection rates elaborated for the five most common brands and pooled for all other brands

	Brand A	Brand B	Brand C	Brand D	Brand E	Other	Total
2018	25.2% (66/262)	15.7% (14/89)	10.8% (8/74)	12.9% (8/62)	0.0% (0/70)	21.4% (12/56)	17.6% (108/613)
2019	24.5% (68/277)	25.3% (20/79)	4.6% (3/65)	1.9% (1/53)	1.5% (1/65)	18.2% (8/44)	17.3% (101/583)
2020	30.4% (111/365)	25.7% (18/70)	6.5% (5/77)	8.7% (4/46)	1.6% (1/64)	21.6% (8/37)	22.3% (147/659)
2021	27.7% (97/350)	20.8% (15/72)	2.3% (3/130)	8.7% (4/46)	1.2% (1/83)	13.5% (7/52)	17.3% (127/733)
Average	27.0%	21.9%	6.0%	8.1%	1.1%	18.7%	18.6%

**Fig. 1:**
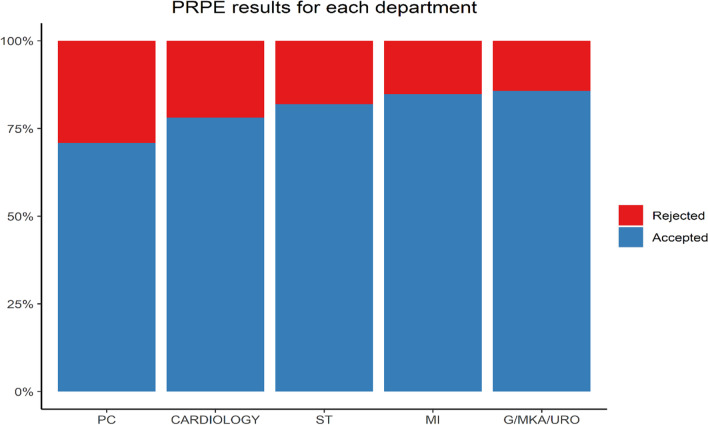
Overview of test results for departments with the highest use of PRPE. Left to right: PC = Pain clinic, Cardiology = Catheterization lab, ST = Surgery theatre, MI = Medical Imaging, G/MKA/URO = Gastroenterology/Oral and maxillofacial surgery/Urology

Figure 1 displays a proportional view of the test results of the departments that rely the most on fluoroscopically guided procedures. Some departments, i.e. emergency room, nuclear imaging and paediatrics, were excluded considering the rarity of PRPE use. Analogously, PRPE belonging to a reserve stock was also excluded.

Table 2 further elaborates on Fig. 1 for the five most frequently used brands and rejection rates combined for the other brands. Department and test results were found to be associated (*p *value < 0.001). The departments (PC and Cardiology) were significantly higher in rejection rate compared to the other departments (*p *value < 0.01).

**Table 2:** Proportional view of the five most common brands per department with global rejection rate

	Brand A	Brand B	Brand C	Brand D	Brand E	Other	Total
PC	34.9% (44/126)	21.3% (10/47)	7.4% (2/27)	0.0% (0/3)	/	45% (9/20)	28.3% (63/223)
Cardiology	35.2% (120/341)	0.0% (0/3)	6.8% (10/252)	14.8% (4/27)	/	6.7% (2/30)	21.9% (143/653)
ST	25.0% (127/509)	21.6% (48/222)	0.0% (0/15)	10.0% (7/70)	1.5% (3/200)	14.6% (7/48)	18.0% (192/1064)
MI	26.2% (37/141)	23.7% (9/38)	0.0% (0/27)	8.5% (4/47)	0.0% (0/82)	17.5% (7/40)	15.2% (57/375)
G/MKA/URO	12.1% (8/66)	/	/	0.0% (0/18)	/	33.3% (7/21)	14.3% (15/105)

Backslashes indicate absence

PC = Pain clinic, Cardiology = Catheterization lab, ST = Surgery theatre, MI = Medical Imaging, G/MKA/URO = Gastroenterology/Oral and maxillofacial surgery/Urology

### Incidence of cracks and tears in repairable PRPE

Of the 766 quality checks performed on repairable pieces, 31.7% (243) led to repairs at least once. Reparation was confirmed in the consecutive year by the health and safety department, and each repaired piece was labelled with a repair note. Almost 50% (117) of the repaired pieces were again rejected in the year following repair.

**Fig. 2:**
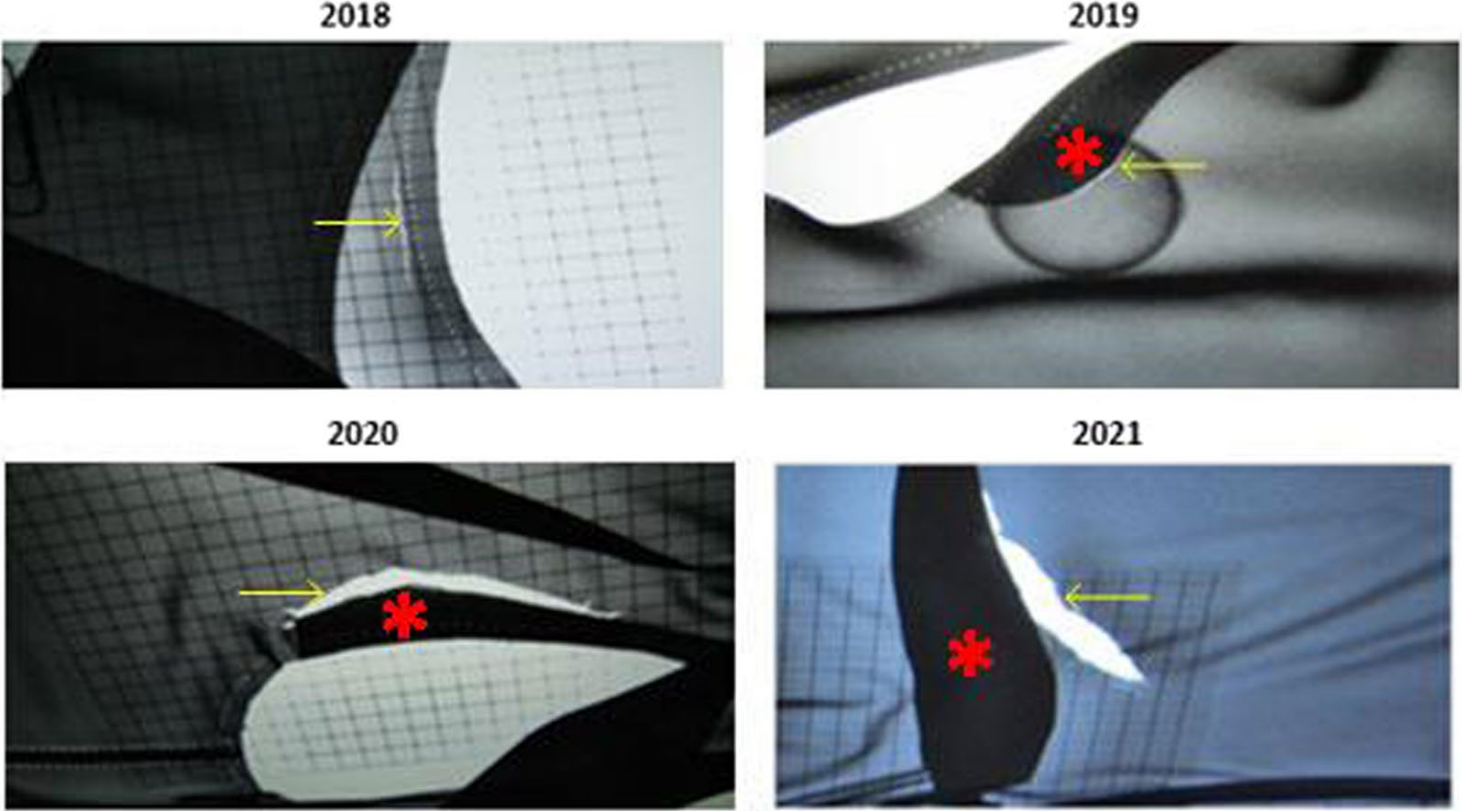
Tear (yellow arrow) evolution in a lead-free vest that was sent yearly to the manufacturer for repair. The X-ray opaque grid to measure the tears is seen in the upper left, bottom left and bottom right picture. The dark patch (red asterisk) is a piece of lead-free fabric that is placed over the tear at the left arm hole. In 2019, a new tear emerged right beside the repair patch. The round sphere shows the tennis ball to identify the tear more easily. In 2020, after two repairs, a big tear of ± 300 mm^2^ is clearly visible. In 2021, after three repairs, another big tear formed beside the repair patch

In detail, 46.7% (28/60) were rejected in both 2018 and 2019, 60% (45/75) were rejected in both 2019 and 2020, and 43% (43/100) were rejected in both 2020 and 2021. These percentages include pieces that were rejected in three and/or four successive years, which were 27 and 5 pieces. Figure 2 displays the evolution of a tear re-occurring after repair in a vest that was rejected in all four years.

### PRPE type comparison

**Fig. 3:**
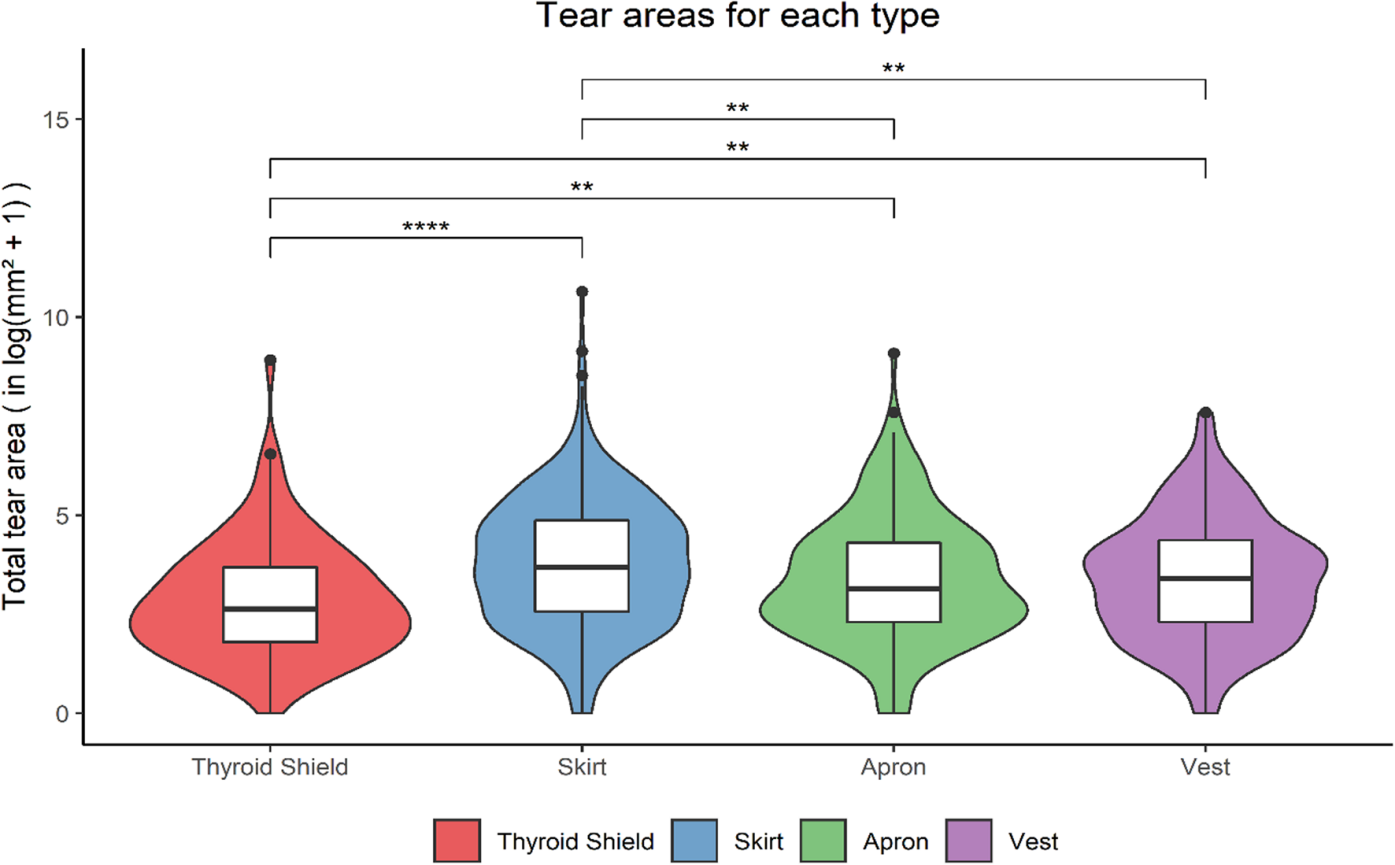
Distribution of tear areas for each type of PRPE on the natural logarithmic scale. The stars indicate statistical significance resulting from Dunn’s post hoc test, and the number of stars indicates the level of statistical significance. Statistically, aprons and vests did not differ significantly in terms of total tear area

Figure 3 shows the distribution of tear areas per PRPE type combined over the study period. The incidence rejections per type were, respectively, 8.1% (69/856), 26.3% (171/649), 19.6% (76/388) and 24% (167/695) for thyroid shields, skirts, aprons and vests. The median tear areas in mm^2^ on the natural logarithmic scale (± interquartile range) were, respectively, 2.7 ± 1.8, 3.1 ± 2.2, 3.2 ± 1.9 and 3.4 ± 2, for thyroid shields, skirts, aprons and vests.

## Discussion

PRPE is crucial in operator radiation protection. Whether non-lead, lightweight PRPE offers the same degree of protection is not always clear [7, 17–23]. Part of this inconsistency in radiation protection might be explained by PRPE having cracks and tears in the attenuating layer. Previous publications briefly mentioned the need for X-ray-based integrity checks of PRPE to assure adequate radiation protection [13, 14, 19, 20, 24, 25]. Still, PRPE data on X-ray-based integrity analysis are very scarce in the literature and limited in terms of tear analysis, sample size and longitudinal follow-up. Furthermore, local legislation is unclear regarding PRPE. The Belgian law states that PRPE should be available and of adequate quality and does not mention frequency nor the methodology of testing PRPE. Therefore, facilities develop their own policies and procedures such as the rejection criteria based on literature and frequency of quality checks. Our study analysed a large sample of longitudinal quantitative quality assurance data on PRPE. Moreover, our study is the first to analyse the progression of tears and the first to investigate the integrity of repaired PRPE.

Our data indicate that more than half of the controlled pieces of PRPE’s revealed tears. This high incidence of tears led to yearly rejections rates between 17 and 22% with an average rejection rate of 18.6%. Despite the hospital’s yearly remedial measures, i.e. discarding or repairing where applicable, the necessity of performing quality checks on PRPE persists in order to assure quality radiation protection. Moreover, it is clear that quality checks are necessary starting from the first day of use. Indeed, our data illustrate that 6.0% of pieces of PRPE pieces showed tears in their first year of use. New garments are hence not guaranteed to remain free of cracks in their first year of use, greatly emphasising the requirement of PRPE quality assurance starting from day one. Similar conclusions were presented by Glaze et al. and Oppliger-Schäfer et al. who mention that new items are not necessarily flawless and could show regions of increased transparency [25]. However, this requirement extends also to the use of repaired PRPE. Repair did not appear to be a durable solution for rejected PRPE. Almost half of the repairable pieces of PRPE were rejected again after repair. Figure 2 demonstrates an example of the reduced efficiency of repair as tears developed alongside repair patches possibly associated with material strain in close adjacency of a repair patch. Further materials research is needed to investigate whether there is a cause–effect relation between repair and adjacent tears.

It is shown that tears differ in area between types of PRPE (Fig. 3). Obviously, thyroid shields, with a median tear area of 14 ± 34 mm^2^, had significantly smaller tears on average as a result of their size, although there are tears that exceeded 160 mm^2^ (above 5 on the y-axis in Fig. 3). Yet, skirts, with a median tear area of 40 ± 117 mm^2^, had larger tears compared to both aprons and vests. Correspondingly, skirts were rejected more on average than the other types. A possible explanation for this difference in mean tear area may be the result of sitting while wearing the skirt. This folds the attenuating layer causing creases and weakening that region. The same holds for wrong storage where certain regions are folded and therefore damaged. Moreover, these regions were found to have larger transmission levels already increasing radiation exposure to the operator even without a clearly visible tear [9, 14, 19, 24, 26]. PRPE should always be stored properly after each procedure to prolong their lifespan and safeguard their integrity.

PRPE layers can consist of a single attenuating layer, while others consist of a bilayer structure, the latter designed to account for fluorescent X-rays [21, 27, 28]. The brands mentioned in Table 2 are all composed of lead-free materials except Brand C (lead-composite). The materials of Brand E were unknown. Whether lead-free, lead-composite or lead is more robust against frequent use is not clear and needs to be further investigated. Additionally, the thickness of the attenuating layer could also be associated with quality control outcomes. In this study, all pieces were 0.25 mm lead equivalent with a wrap-around fit to provide 0.5 mm lead equivalent protection at the front side. Exceptions were thyroid shields and older pieces consisting only of front protection. Both were 0.5 mm lead equivalent. An exact material analysis considering lead equivalency and primary substances making up the lead equivalency could provide additional information on how the attenuating layer would react to frequent use [26, 29].

Present study has some limitations. Although the discrepancies in tear areas between types of PRPE were statistically significant, it is important to acknowledge the measurement uncertainty of our method. The use of an X-ray opaque grid is not ideal to estimate tear areas because tears are irregular in shape making it erroneous to estimate tear areas using a straight ruler with an accuracy of 0.5 cm. Also, the use of a tennis ball (Fig. 2) in certain situations could have overestimated the tear. Yet, without using a tennis ball, there was a risk of missing a tear, perhaps accepting a piece that would otherwise be rejected. Nonetheless, the differences between types of PRPE would persist regardless of measurement error. Besides the difficulty of estimating tear areas, X-ray-based integrity analysis is very laborious and time-consuming as a piece is typically larger than the radiation field of a tele-operated X-ray table. This obliges the inspectors to scan each piece region by region.

Moreover, differences in rejection rates between brands cannot be caused solely by brand difference. The incidence of tears in certain brands should be examined for the type of department where PRPE is used. Departments differ in terms of PRPE use, simply due to the differences in procedures such as duration and operator movement during the procedure. Indeed, medical imaging procedures are often conducted from behind a lead screen, eliminating the need for PRPE. On the contrary, operators in surgery theatres and interventional departments have to be in close proximity to the patient, simply due to the nature of interventions, demanding more intensive use of PRPE [1, 2, 5, 30]. Still our data suggest a positive correlation between tear incidence and departments requiring regular and intensive use of PRPE. Similar conclusions were presented by Bawazeer et al., Oppliger-Schäfer et al. and Finnerty et al. [13, 14, 20]. Unfortunately, determining the exact relation between PRPE use and tear incidence for each PRPE garment is not straightforward, mainly because of the multiple confounding factors lurking behind PRPE use and tear incidence. Such factors are duration of use, intensity of use and whether or not multiple persons wear the same garment. Remarkably, only a minor fraction of the PRPE was bought for an individual user (14.9%) and hence has a correct fit for that person. The vast majority of pieces of PRPE were available for several users. The right fit is important to lower the risk of unnecessary tension on the garment and ensure adequate shielding efficacy [26, 31]. Consequently, this hindered an analysis of the association between tears and the clinical tasks performed by the corresponding user. Besides departmental and user discrepancies, tear incidence could be affected by differences in attenuating layer materials.

## Conclusions

PRPE has to be inspected regularly under X-ray examination upon receipt. New and repaired pieces are not guaranteed to remain free of cracks and tears. Most rejections were found in departments that heavily rely on fluoroscopy due to the nature of their procedures. Whether their increased rejection rates are caused solely by the intensity of use has to be further investigated. Probably, the composition of the attenuating layer could alter a certain piece more or less sensible to tears, particularly in combination with intensive use. It is, however, premature to make such inferences in this study. In future material analyses, the effect of movement, sitting and abusive storage should be addressed. These could cause creases and increase transparency in that region possibly increasing X-ray exposure. Finally, a Monte Carlo-based study could provide more precise estimates of the true additional absorbed dose of X-rays due to a tear [26, 32, 33]. These results might imply other rejection criteria substantiated by dosimetry instead of the economically derived criteria from Lambert & McKeon [15, 34].

## Data Availability

The datasets used and analysed during the current study are available from the corresponding author on reasonable request.

## Abbreviations

G/MKA/URO: Gastroenterology/oral and maxillofacial surgery/urology
MI: Medical imaging
PC: Pain clinic
PRPE: Personal radiation protective equipment
ST: Surgery theatre
TN: Technical number
